# Supplementary material for: The conformational landscape of fold-switcher KaiB is tuned to the circadian rhythm timescale
Source: bioRxiv. 2024 Jun 3:2024.06.03.597139. Preprint. [Version 1] doi: 10.1101/2024.06.03.597139 (PMC11185700; doi:10.1101/2024.06.03.597139)
Supplement: Supplementary file 1 [file media-1.pdf]

Supporting Information for
The conformational landscape of fold-switcher KaiB is tuned to the circadian rhythm timescale

Hannah K. Wayment-Steele*, Renee Otten*, Warintra Pitsawong*, Adedolapo M. Ojoawo, Andrew Glaser, Logan A. Calderone, Dorothee Kern. *These authors contributed equally.

Dorothee Kern
Email: dkern@brandeis.edu

This PDF file includes:

Figures S1 to S11
Tables S1 to S2
SI References

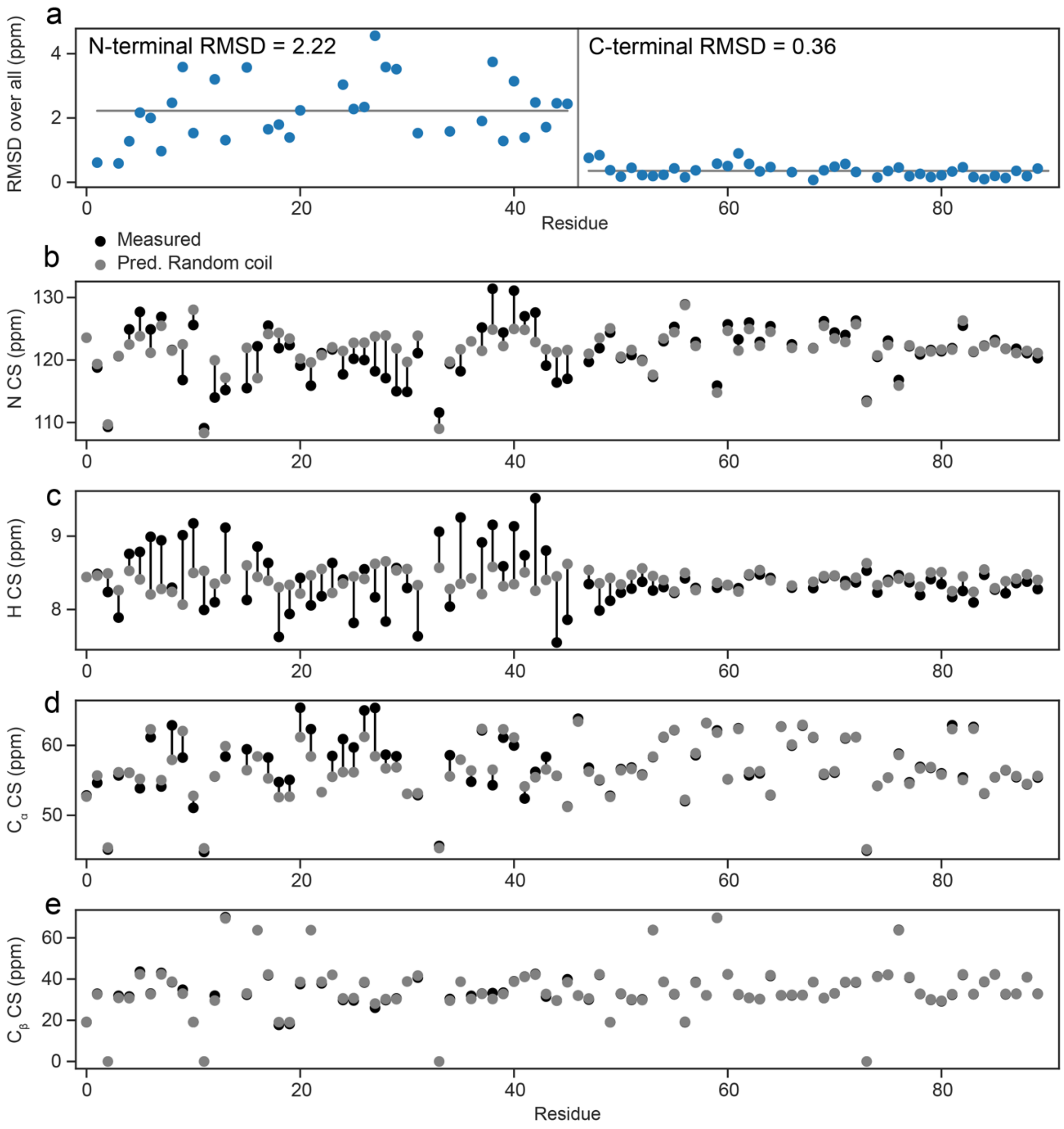

Fig. S1. Major state at 4˚C is a partially disordered state. Chemical shifts of C-terminus (Leu46 onwards) for major state at 4˚C correlate well with random coil chemical shifts predicted by POTENCI[1]. (a) RMSD per residue of N, H, Cα, Cβ chemical shifts relative to random coil shifts. (b-e) Measured (black) vs. predicted (grey) for random coil chemical shifts for N, H, Cα, Cβ atoms.

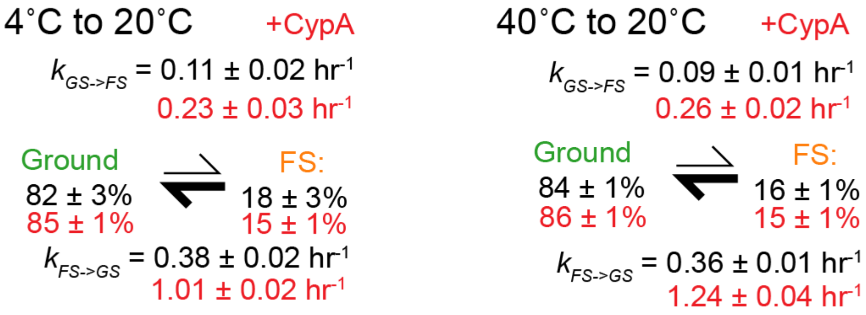

Fig. S2. Kinetics for Ground to FS interconversion and rate acceleration by CypA. Rate constants and populations fit from KaiB incubated at 4˚C (left) or 40 ˚C (right) and monitored at 20˚C (compare to Fig. 1b).

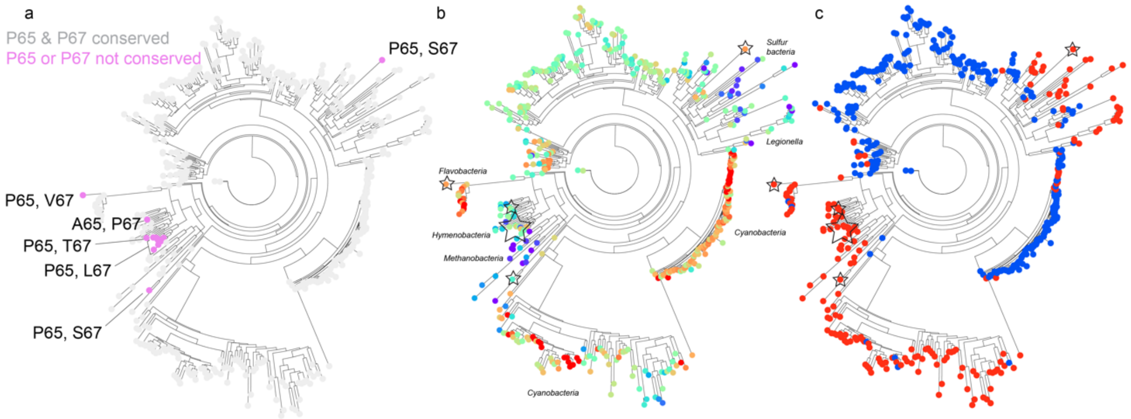

Figure S3. Conservation of P65 and P67 across KaiB phylogenetic tree in ref. [2]. a) Sequences in grey have P65 and P67 conserved. Sequences in magenta have only one of these two prolines, mutations noted on figure. b) Tree colored by pLDDT from closest-10 AF2 predictions and c) Predicted FS (red) or Ground state (blue) from ref. [2].

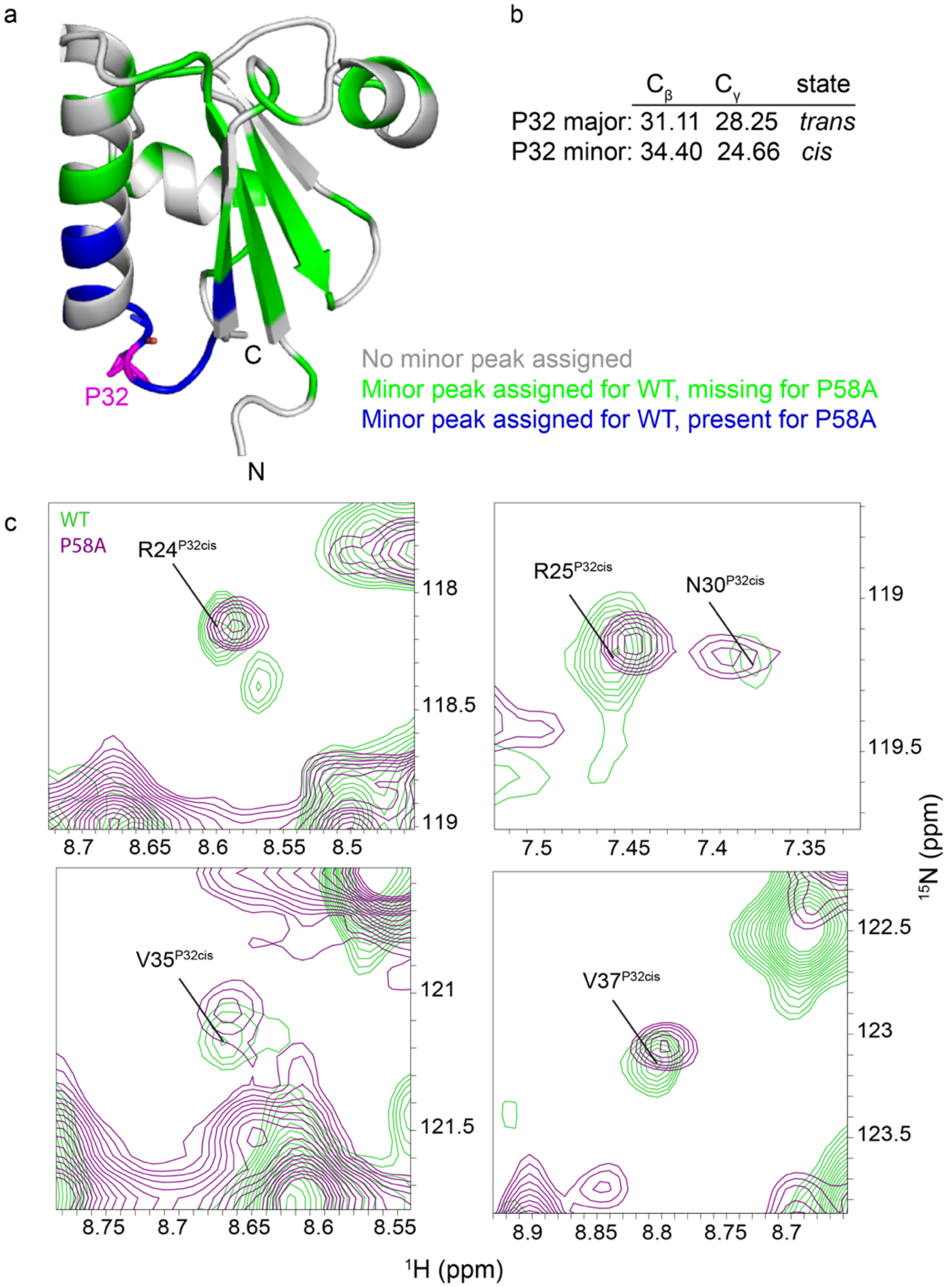

Fig. S4. Some minor peaks correspond to a minor *cis* state of P32. a) Fold-switched structure of KaiB from AF-Cluster, colored by grey: no minor peak could be assigned, green: a minor peak was assigned in KaiB WT but not in KaiB P58A, blue: a minor peak was assigned in both KaiB WT and KaiB P58A. b) The C_β and C_γ shifts of the major and minor peak of P32 correspond to a trans and cis state. c) representative minor peaks present for both KaiB WT (green) and KaiB P58A (purple).

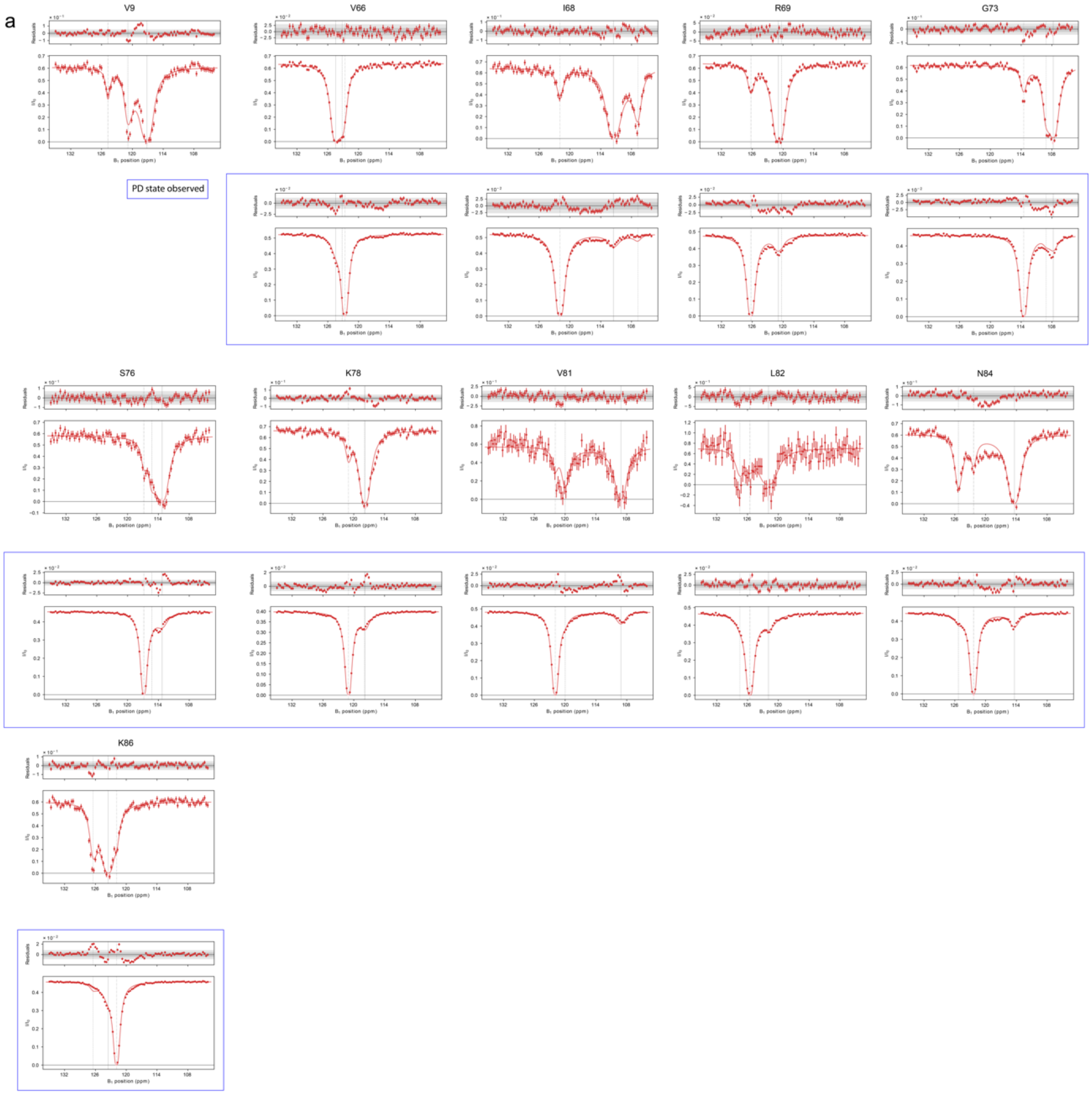
a
V9
V66
I68
R69
G73
PD state observed
S76
K78
V81
L82
N84
K86
Residuals
B₁ position (ppm)

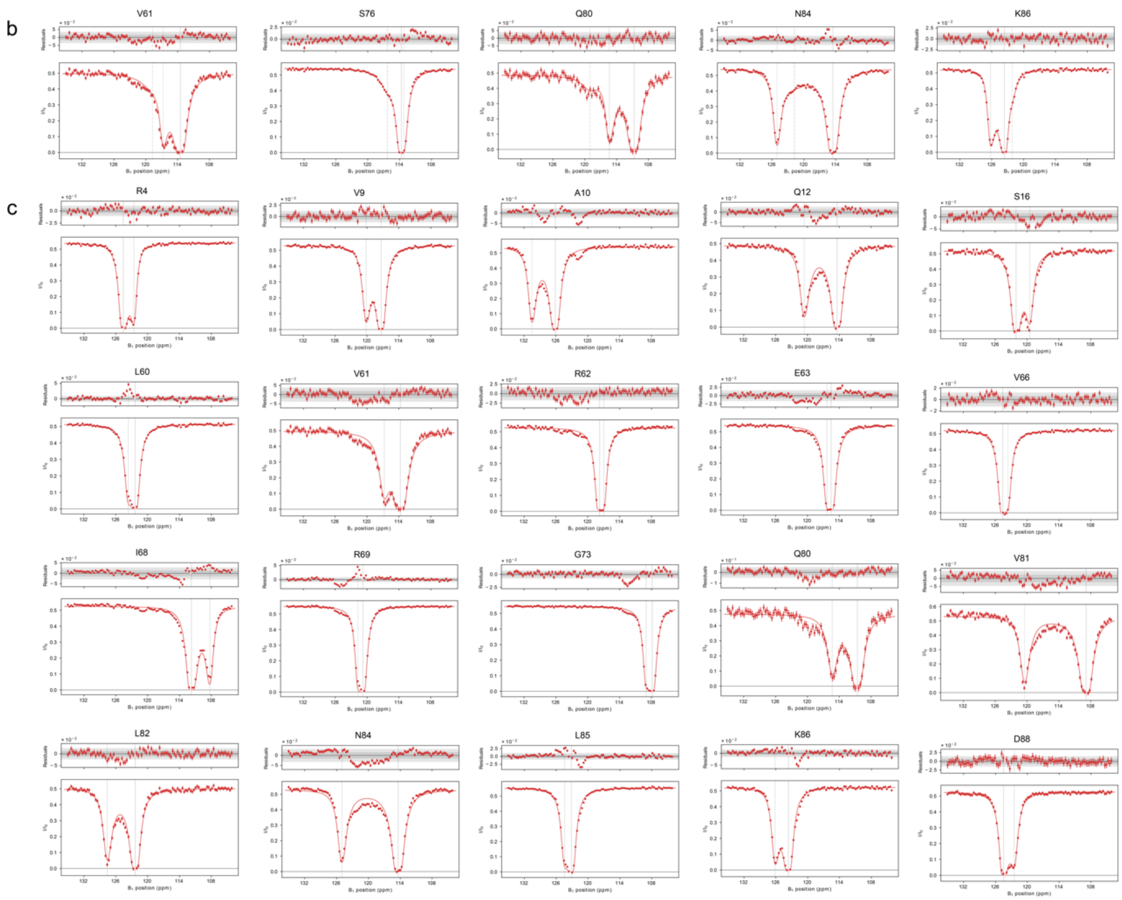

Fig. S5 (multi-page figure). **^{15}N CEST data overlaid with global fits** for (a) 3-state fit at 4˚C (PD state observed is boxed in blue), (b) 3-state fit at 20˚C, (c) 2-state fit to Ground and Enigma state at 20˚C.

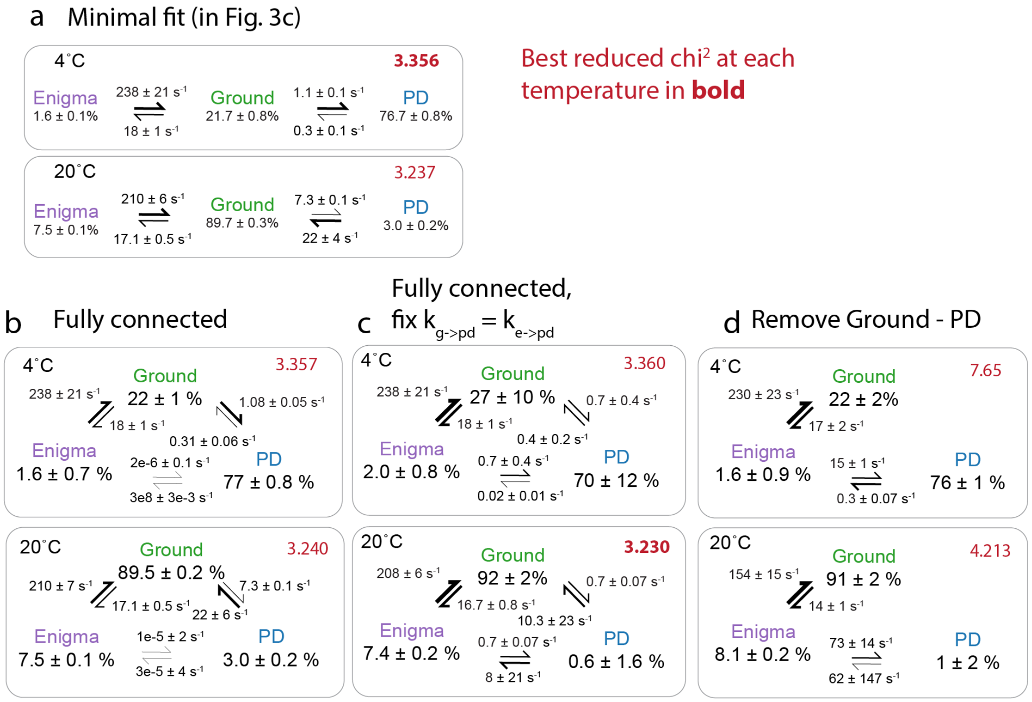

Fig. S6. Evaluating alternate models for fitting CEST data. (a) Minimal models representing data, reproduced from Fig. 3c. (b) Allowing all states to interconvert. (c) Constraining $k_{Ground\text{->}PD} = k_{Enigma\text{->}PD}$. (d) Removing the Ground-PD interconversion results in notably worse models by reduced Chi^2. Taken together, this suggests that there could be even up to equal flux to the PD state through the enigma state as directly from the Ground state. The enigma to PD interconversion is underdetermined because of the too low populations. Physically, it would make most sense if both GS and enigma can interconvert with the PD state given their similar structures. These alternate fits rule out the path going exclusively through the Enigma state.

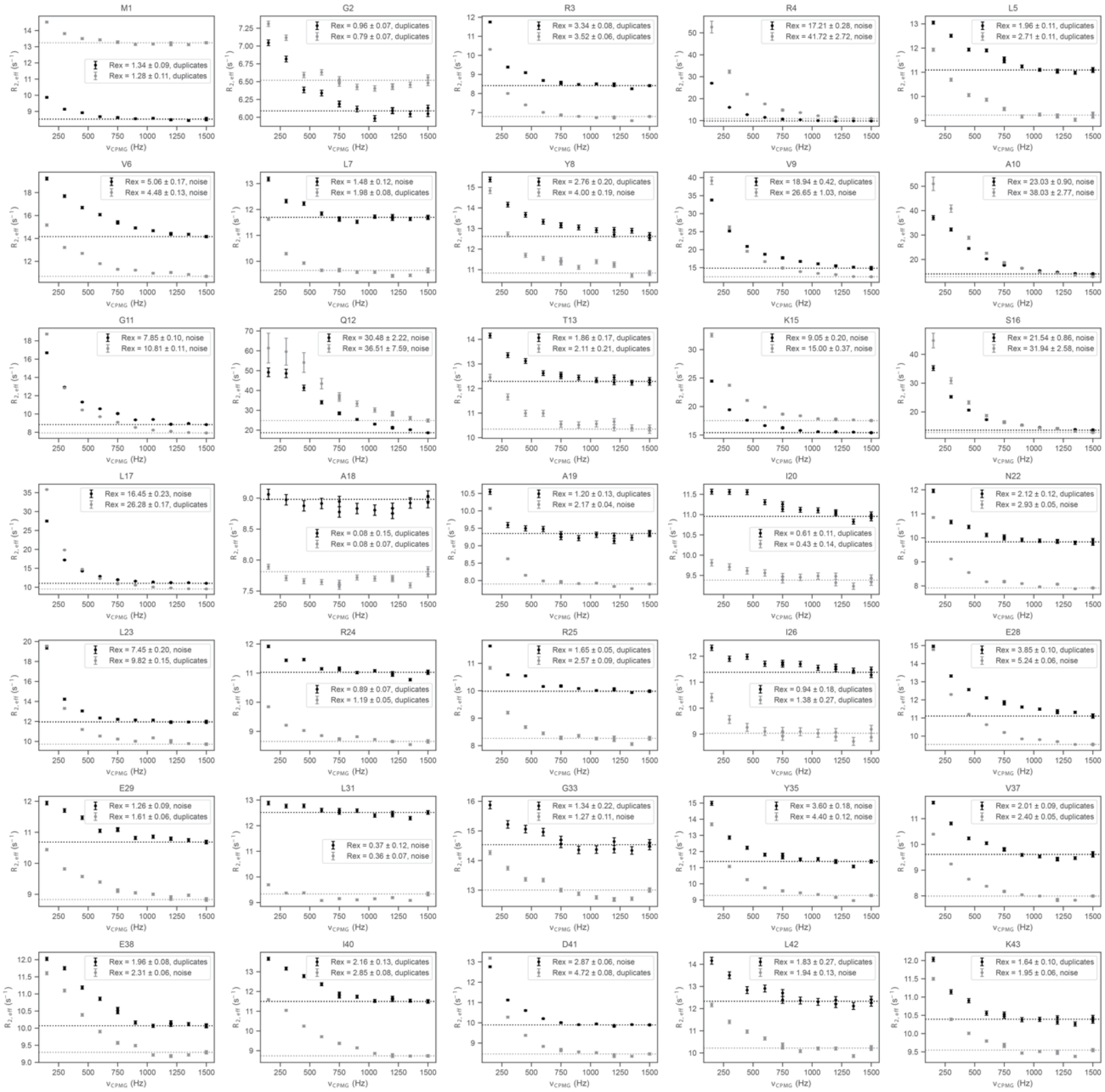

Fig. S7. (multi-page figure, 1 of 2). ^{15}N CPMG data for all assigned Ground state peaks at 25˚C and 35˚C.

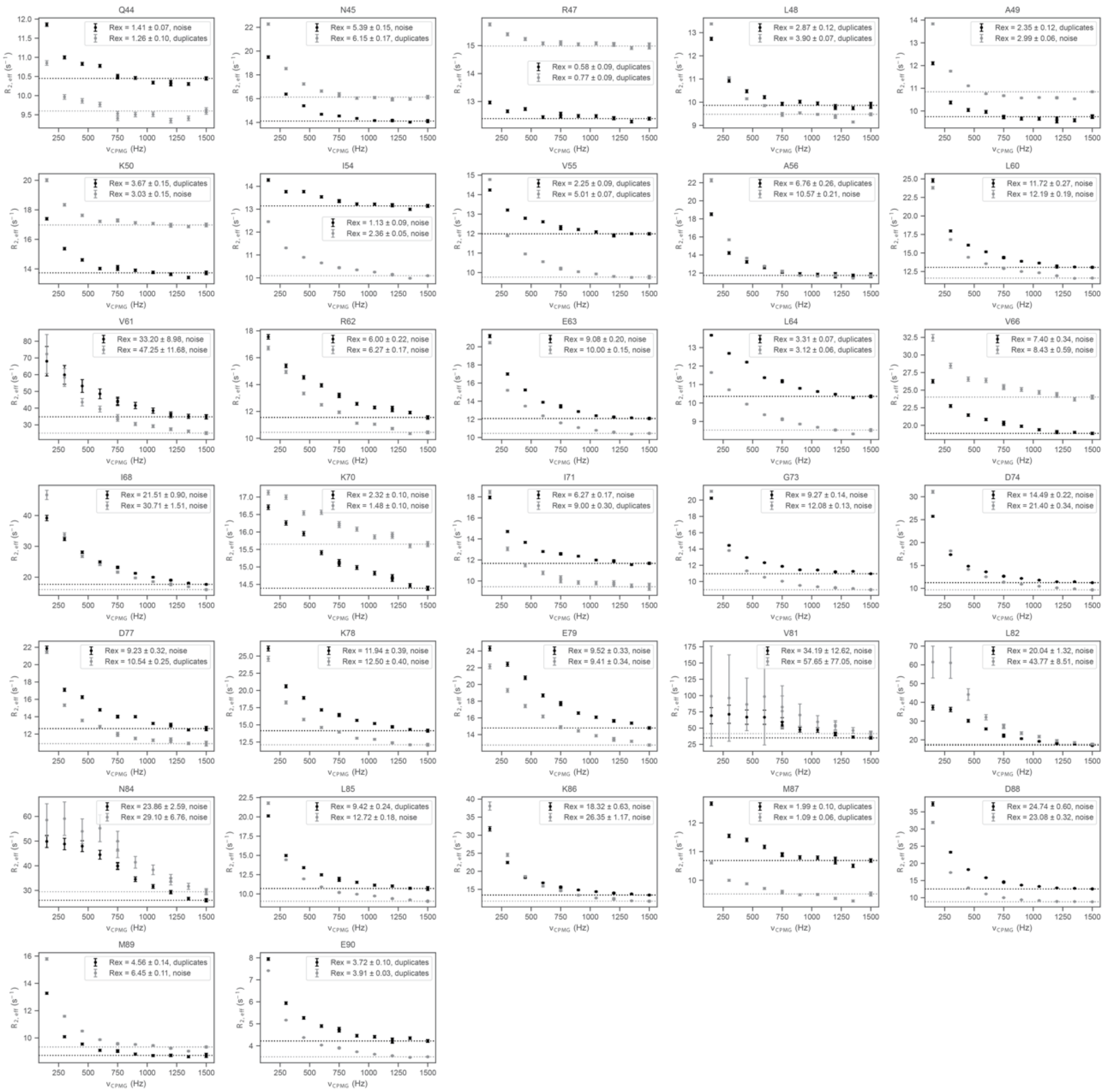

Fig. S7. (multi-page figure, 2 of 2). ^{15}N CPMG data for all assigned Ground state peaks at 25˚C and 35˚C.

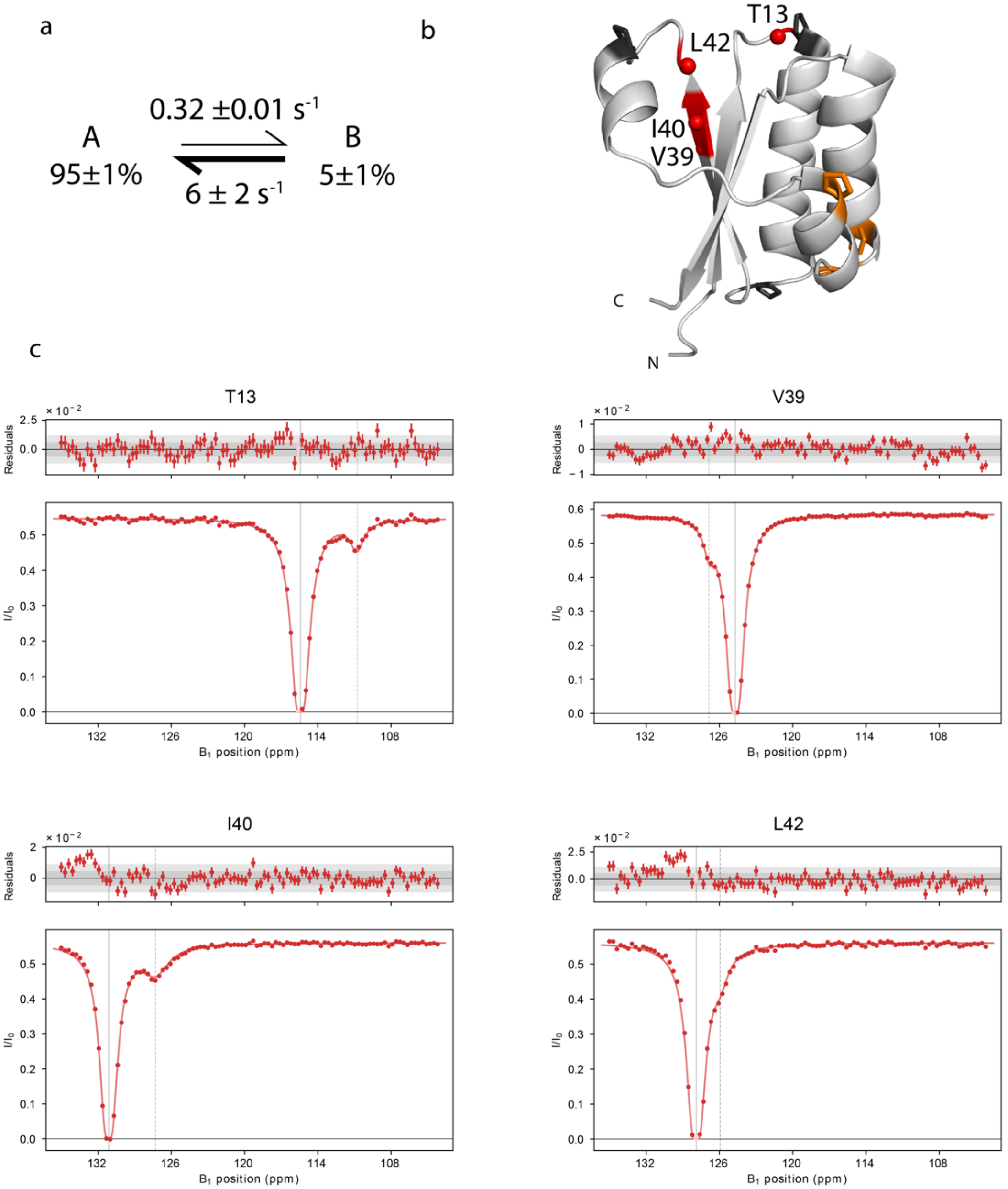

Fig. S8. Different minor slow process fit in CEST data at 20˚C, likely due to cis/trans prolyl isomerization of Pro14. a) Populations and kinetics from 2-state fit. b) Location of residues fit to process visualized in red. Prolines P14, P32, P48 shown in black. Prolines P58, P65, P67, which are *trans* in the Ground and *cis* in the FS state, are shown in orange. c) CEST data from field strength = 20 Hz overlaid with fits from ChemEx.

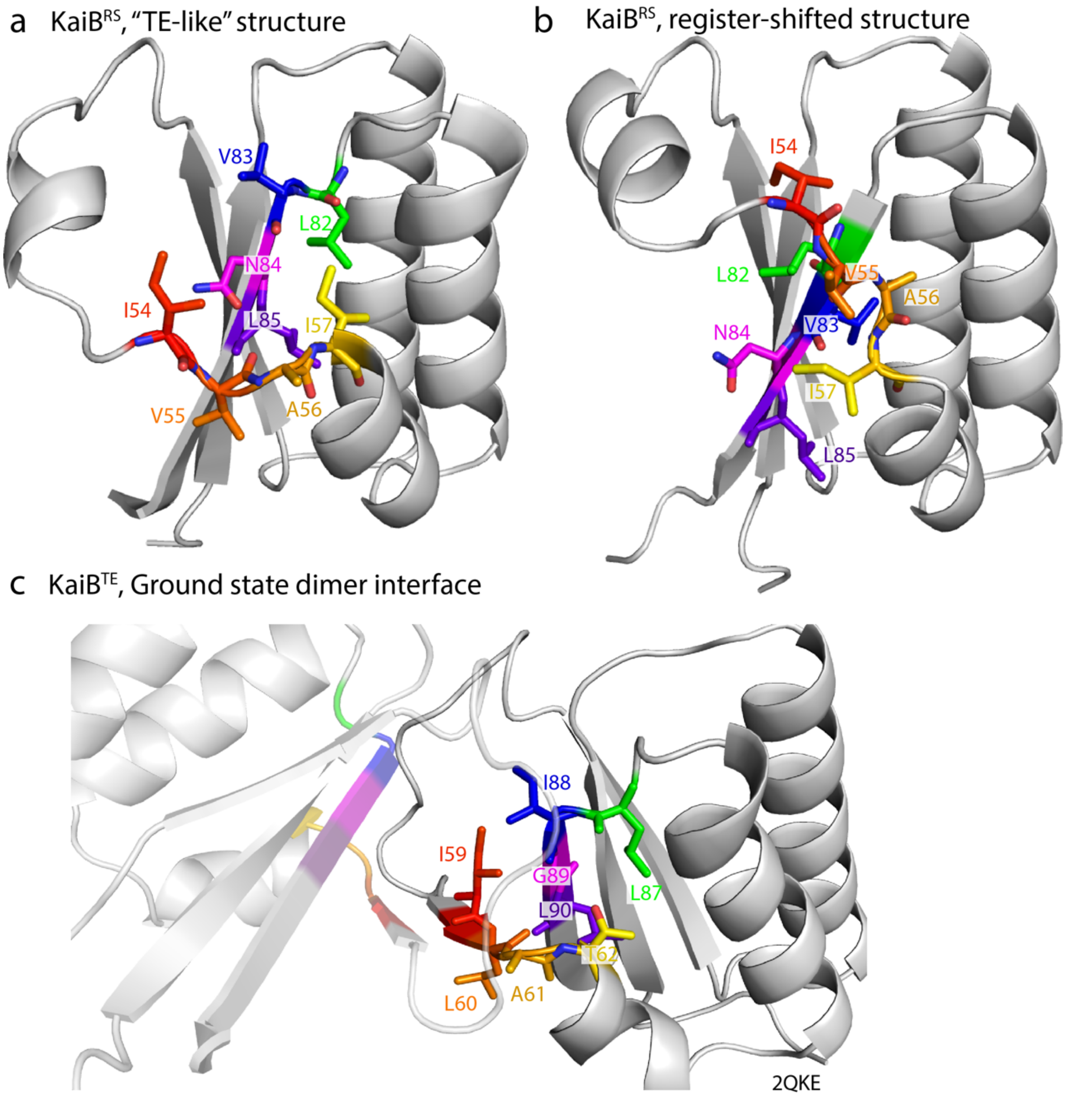

Figure S9. Difference in hydrophobic packing of core between (a) TE-like and (b) register-shifted, proposed Enigma structure. (c) The hydrogen-bonding and hydrophobic core of the TE-like structure is homologous to the crystal structure of KaiB from *Thermosynechococcus elongatus* (PDB: 2QKE [ref]). I59, L60, A61 are involved in the dimerization interface in the tetrameric structure in *T. elongatus*, and this same geometry is predicted for homologous residues in the TE-like structure of *R. sphaeroides* (compare to (a)).

TE-like structure: 5/6 observable NOE crosspeaks observed

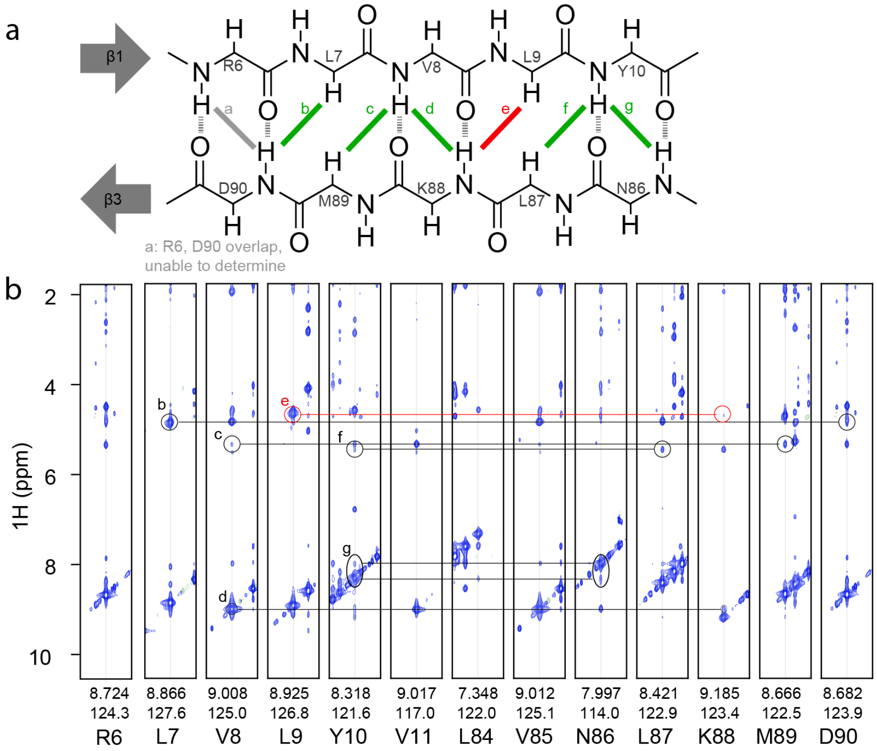

Fig. S10. NOESY data supporting the TE-like structure as the major ground state observed via NMR. (a) cartoon of hydrogen bonding corresponding to TE-like structure, for which 5/6 observable NOEs had support, annotated on NOESY strip plot in (b). Green indicates NOEs which have support, red indicates NOEs which do not have support. One NOE between R6 and D90 was unable to be confirmed or denied due to overlap.

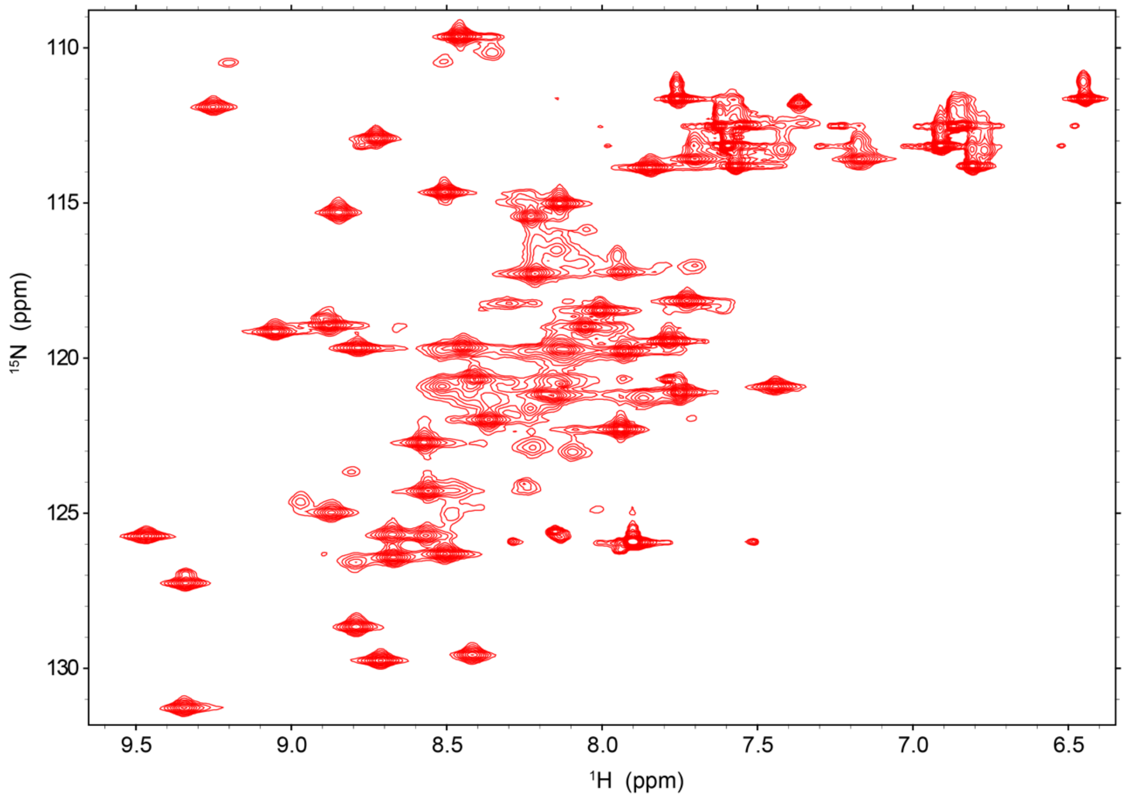

Figure S11. MiniKaiB is folded over temperature range from 4 ˚C to 25 ˚C. ^{1}H-^{15}N HSQC of KaiB-ΔCterm at 25˚C.

Table S1. Proline isomerization states in homologous structures of KaiB Ground and FS state. "—"= proline not conserved.

State	PDB code	Method	Organism / notes	Reference	T13-P14	L31-P32	N45-P46	I57-P58	L64-P65	V66-P67
Ground	2QKE	X-ray	*T. elongatus*	Pattanayek et al. (2008)[3]	*trans*	*trans*	*trans*	*trans*	*trans*	*trans*
Ground	n/a	NMR	*R. sphaeroides*	This work	*trans*	*trans/cis*	*trans*	*trans*	*trans*	*trans*
FS	4KUN	X-ray	*L. pneumophila*	Loza-Correa et al. (2014)[4]	--	*trans*	--	*cis*	*cis*	*cis*
FS	5JYT	NMR	*T. elongatus* + mutations	Tseng et al. (2017)[5]	*trans*	--	*trans*	*cis*	*cis*	*cis*
FS	8FWJ	Cryo-EM	*R. sphaeroides*	Pitsawong et al. (2023)[6]	*trans*	*trans*	*trans*	*cis*	*cis*	*cis*
FS	8UBH	NMR	*T. elongatus vestitus,* KaiB-4	Wayment-Steele et al. (2024)[2]	--	*trans*	*trans*	*cis*	*cis*	*cis*
FS	n/a	NMR	*R. sphaeroides*	This work	*trans*	*trans/cis*	*trans*	*cis*	*cis*	*cis*
PD	n/a	NMR	*R. sphaeroides*	This work	*trans*	*trans/cis*	*trans*	*trans*	*trans*	*trans*

Supplemental Data 1. Energetic contributions for TE-like and register-shifted structures estimated with FoldX[7].

Supplemental Data 2. Estimated R_{ex} and determined exchange regime from CPMG at 25˚C and 35˚C.

Supplemental Data 3. Calculations pertaining to barrier heights and populations of all states for KaiB depicted in Fig. 5.

SI References

1 Nielsen, J. T. & Mulder, F. A. A. POTENCI: prediction of temperature, neighbor and pH-corrected chemical shifts for intrinsically disordered proteins. *J Biomol NMR* **70**, 141-165 (2018). https://doi.org:10.1007/s10858-018-0166-5

2 Wayment-Steele, H. K. *et al.* Predicting multiple conformations via sequence clustering and AlphaFold2. *Nature* **625**, 832-839 (2024). https://doi.org:10.1038/s41586-023-06832-9

3 Pattanayek, R. *et al.* Structural model of the circadian clock KaiB-KaiC complex and mechanism for modulation of KaiC phosphorylation. *EMBO J* **27**, 1767-1778 (2008). https://doi.org:10.1038/emboj.2008.104

4 Loza-Correa, M. *et al.* The Legionella pneumophila kai operon is implicated in stress response and confers fitness in competitive environments. *Environ Microbiol* **16**, 359-381 (2014). https://doi.org:10.1111/1462-2920.12223

5 Tseng, R. *et al.* Structural basis of the day-night transition in a bacterial circadian clock. *Science* **355**, 1174-1180 (2017). https://doi.org:10.1126/science.aag2516

6 Pitsawong, W. *et al.* From primordial clocks to circadian oscillators. *Nature* **616**, 183-189 (2023). https://doi.org:10.1038/s41586-023-05836-9

7 Schymkowitz, J. *et al.* The FoldX web server: an online force field. *Nucleic Acids Res* **33**, W382-388 (2005). https://doi.org:10.1093/nar/gki387
